# Stimulation-Based Control of Dynamic Brain Networks

**DOI:** 10.1371/journal.pcbi.1005076

**Published:** 2016-09-09

**Authors:** Sarah Feldt Muldoon, Fabio Pasqualetti, Shi Gu, Matthew Cieslak, Scott T. Grafton, Jean M. Vettel, Danielle S. Bassett

**Affiliations:** 1 Department of Bioengineering, University of Pennsylvania, Philadelphia, Pennsylvania, United States of America; 2 US Army Research Laboratory, Aberdeen Proving Ground, Maryland, United States of America; 3 Department of Mathematics and Computational and Data-Enabled Science and Engineering Program, University at Buffalo, SUNY, Buffalo, New York, United States of America; 4 Department of Mechanical Engineering, University of California, Riverside, Riverside, California, United States of America; 5 Applied Mathematics and Computational Science Graduate Program, University of Pennsylvania, Philadelphia, Pennsylvania, United States of America; 6 Department of Psychological and Brain Sciences, University of California, Santa Barbara, Santa Barbara, California, United States of America; 7 Department of Electrical and Systems Engineering, University of Pennsylvania, Philadelphia, Pennsylvania, United States of America; Hamburg University, GERMANY

## Abstract

The ability to modulate brain states using targeted stimulation is increasingly being employed to treat neurological disorders and to enhance human performance. Despite the growing interest in brain stimulation as a form of neuromodulation, much remains unknown about the network-level impact of these focal perturbations. To study the system wide impact of regional stimulation, we employ a data-driven computational model of nonlinear brain dynamics to systematically explore the effects of targeted stimulation. Validating predictions from network control theory, we uncover the relationship between regional controllability and the focal versus global impact of stimulation, and we relate these findings to differences in the underlying network architecture. Finally, by mapping brain regions to cognitive systems, we observe that the default mode system imparts large global change despite being highly constrained by structural connectivity. This work forms an important step towards the development of personalized stimulation protocols for medical treatment or performance enhancement.

## Author Summary

Brain stimulation is increasingly used in clinical settings to treat neurological disorders, but much remains unknown about how stimulation to a single brain region impacts large-scale, brain network activity. Using structural neuroimaging scans, we create computational models of brain dynamics for eight participants to explore how structure-function relationships constrain the effect of stimulation to a single region on the brain as a whole. Our results show that network control theory can be used to predict if the effects of stimulation remain focal or spread globally, and structural connectivity differentially constrains the effects of regional stimulation. Additionally, we study how stimulation of different cognitive systems spreads throughout the brain and find that stimulation of regions within the default mode network provide a mechanism to impart large change in overall brain dynamics through a densely connected structural network. By revealing how the stimulation of different brain regions and cognitive systems spreads differently through the brain, we provide a modeling framework to develop stimulation protocols to personalize medical treatments, enable performance enhancements, and facilitate cortical plasticity.

## Introduction

Brain stimulation is increasingly used to diagnose [1], monitor [2], and treat neurological [3] and psychiatric [4] disorders. Non-invasive stimulation, such as transcranial magnetic stimulation (TMS) or transcranial direct current stimulation (tDCS), is used, for example, in epilepsy [5, 6], stroke [7], attention deficit hyperactivity disorder [2], tinnitus [8], headache [9], aphasia [10], traumatic brain injury [11], schizophrenia [12], Huntington’s disease [13], and pain [14], while invasive deep brain stimulation (DBS) is approved for essential tremor and Parkinson’s disease and is being tested in multiple Phase III clinical trials in major depressive disorder, Tourette’s syndrome, dystonia, epilepsy, and obsessive-compulsive disorder [15]. In addition to its clinical utility, emerging evidence suggests that stimulation can also be used to optimize human performance in healthy individuals [16, 17], potentially by altering cortical plasticity [17].

Despite its broad utility, many engineering challenges remain [18], from the optimization of stimulation parameters to the identification of target areas that maximize clinical utility [19]. Critically, an understanding of the local effects of stimulation on neurophysiological processes—and the downstream effects of stimulation on distributed cortical and subcortical networks—remains elusive [20]. This gap has motivated the combination of stimulation techniques with various recording devices (PET [21], MEG [22], fast optical imaging [23], EEG [17], and fMRI [24, 25]) to monitor the effects of stimulation on cortical activity [26–28]. In this context, it has become apparent that there is a critical need for biologically informed computational models and theory for predicting the impact of focal neurostimulation on distributed brain networks, thereby enabling the generalization of these effects across clinical cohorts as well as the optimization of stimulation protocols [29], including refinements for individualized treatment and personalized medicine.

To meet this fast-growing need, we utilize network control theory [30] to understand and predict the effects of stimulation on brain networks. The advent of network theory as a ubiquitous paradigm to study complex engineering systems as well as social and biological models has changed the face of the field of automatic control and redefined its classic application domain. *Control* of a network refers to the possibility of manipulating local interactions of dynamic components to steer the global system along a chosen trajectory. Network control theory offers a mechanistic explanation for how specific regions within well-studied cognitive systems may enable task-relevant neural computations: for example, activity in the primary visual cortex can initiate a trajectory of processing along the extended visual pathway (V2, V3, etc.), driving visual perception and scene understanding. More broadly, network control theory also offers a mechanistic framework in which to understand clinical interventions such as brain stimulation, which elicits a strategic functional effect within the network for synchronized neural processing. In both cases (regional activity and stimulation), it is critically important to understand how underlying structural connectivity can constrain or modulate the functional effect of regional alterations in activity. Yet, the application of control-theoretic techniques to brain networks is underexplored, even though the questions posed by the neuroscientific community are uniquely suited to the application of these tools [30].

In a novel approach to link network control theory and brain dynamics, we examine the effects of regional stimulation on brain states using a nonlinear meso-scale computational model, built on data-driven structural brain networks. We demonstrate that the dynamics of our model are highly variable across subjects, but highly reproducible across multiple scans of the same subject. We confirm the pragmatic utility of network control theory for nonlinear systems, extending previous work on linear approaches [30], and show that general control diagnostics (average and modal controllability) are strongly correlated with the density of structural connections linking brain regions. Finally, we investigate the interplay between functional and structural effects of stimulation by examining how the global functional activity across brain regions is modulated by region-specific stimulation (a region’s functional effect) and whether the region’s structural connectivity accounts for its influence on the larger brain network (structural effect). Results show that the default mode system and subcortical regions produce the strongest functional effects; the subcortical structures display weak structural effects, being diversely connected across many cognitive systems. Collectively, our results indicate the value of data-driven, biologically motivated brain models to understand how individual variability in brain networks influences the functional effects of region-specific stimulation for clinical intervention or cognitive enhancement.

## Results

**Fig 1:**
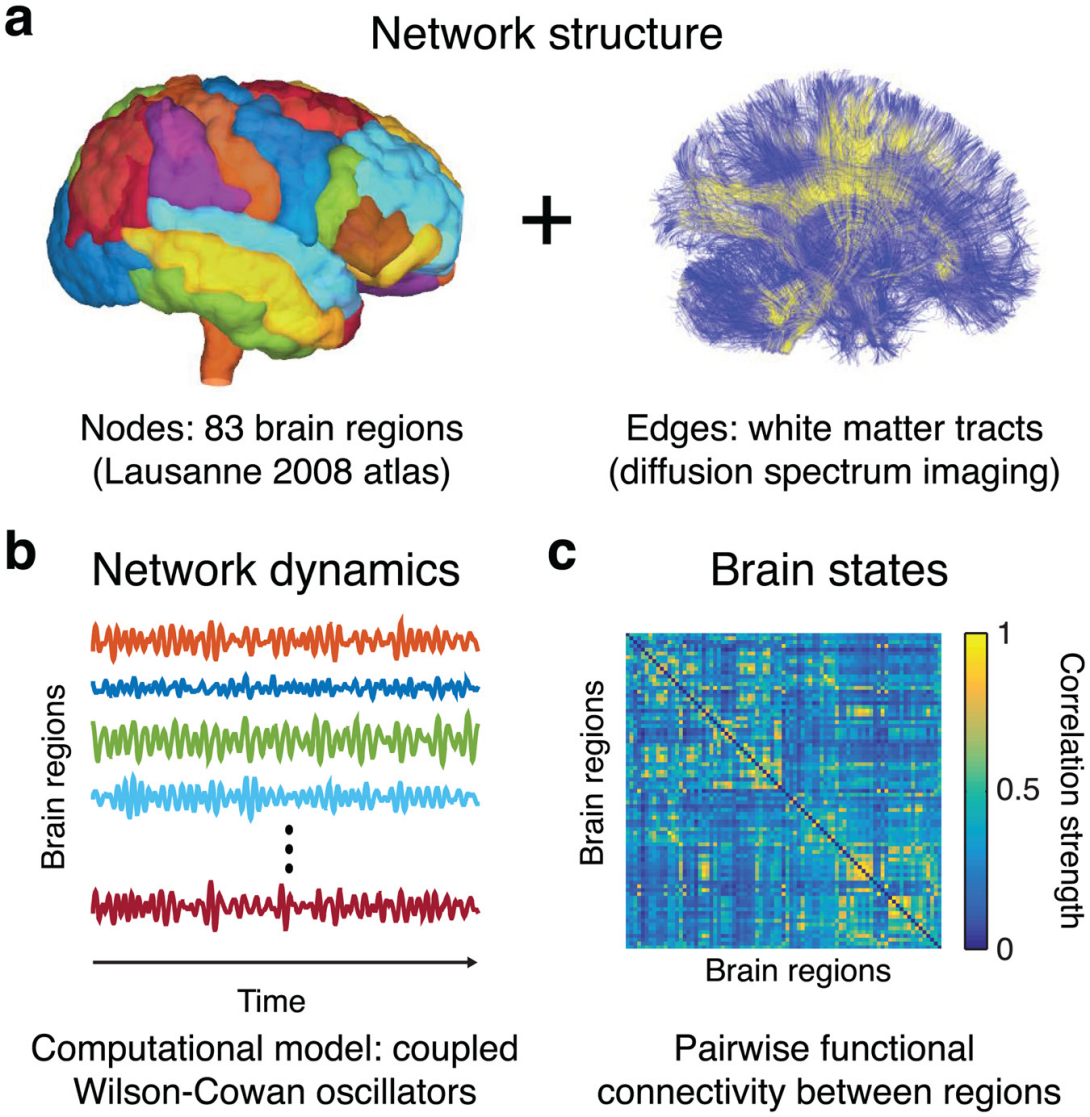
Building nonlinear brain networks. (**a**) Subject-specific structural brain networks are built based on a parcellation of the brain into 83 anatomically defined brain regions (network nodes) with connections between regions given by the density of streamlines linking them. (**b**) The dynamics of each region are represented by a single Wilson-Cowan oscillator, and these oscillators are coupled according to the structural connectivity of a single subject. (**c**) Brain states are quantified by calculating the pairwise functional connectivity between brain regions.

To systematically assess the effects of brain stimulation and its utility in control, we begin by building a spatially embedded nonlinear model of brain network dynamics. Structural brain networks are derived from diffusion spectrum imaging (DSI) data acquired in triplicate from 8 healthy adult subjects. We perform diffusion tractography to estimate the number of streamlines linking *N* = 83 large-scale cortical and subcortical regions extracted from the Lausanne atlas [31] and summarize these estimates in a weighted adjacency matrix whose entries reflect the density of streamlines connecting different regions. Finally, we model regional brain activity using biologically motivated nonlinear Wilson-Cowan oscillators [32], coupled through these data-derived structural brain networks. We quantify brain states based on functional connectivity obtained from pairwise correlations between simulated regional brain dynamics (Fig 1 and Methods).

### Inter-subject variability of brain dynamics

**Fig 2:**
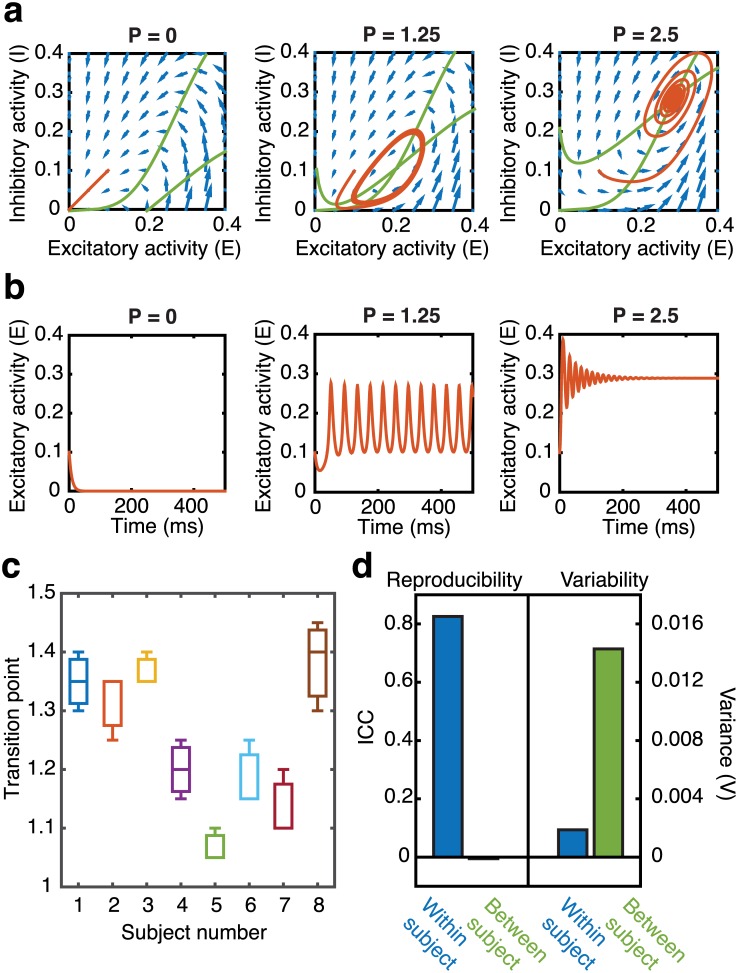
Nonlinear brain dynamics and variability. (**a**) Excitatory-inhibitory phase space plots depicting behavior for a single Wilson-Cowan oscillator in the presence of no external current input (left; *P* = 0; low-fixed point), moderate external current input (middle; *P* = 1.25; limit cycle), and high external current input (right; *P* = 2.5; high fixed point). All simulations are started with initial conditions *E* = 0.1, *I* = 0.1 and nullclines are plotted in green. (**b**) The corresponding firing rate of the excitatory population plotted as a function of time for the simulations depicted in *(a)*. (**c**) Box plots showing the value of global coupling parameter at which the system transitions from the low fixed-point state to the oscillatory regime for models derived from three different structural scans from each of eight subjects. (**d**) Within and between subject reproducibility (left) and variability (right) for the data shown in *(c)*. Reproducibility is measured by the intraclass correlation coefficient (ICC) and is high within subjects indicating a high level of reproducibility between scans of a single subject, but low between subjects indicating a low reproducibility between scans of different subjects. This is additionally reflected in the low within subject variability, measured as the average variance, and high between subject variability.

Our broad goal is to understand the role of regional stimulation to differentially control brain dynamics. Theoretical predictions of regional controllability can be developed based on the underlying structural connectivity of the computational model [30]. Given our subject-specific data-driven approach, it is therefore important to understand how variability between structural brain networks affects the dynamics of our model. Wilson-Cowan oscillators are a biologically driven mathematical model of the mean-field dynamics of a spatially localized population of neurons [32, 33], modeled through equations governing the firing rate of coupled excitatory (*E*) and inhibitory (*I*) neuronal populations (Methods). Here, we measure a single brain region’s dynamics by the firing rate of the excitatory population. An important feature of these Wilson-Cowan oscillators is that an uncoupled oscillator can exhibit one of three states, depending upon the amount of external current applied to the system (Fig 2a and 2b). When no external current is applied (*P* = 0), the system relaxes to a low fixed point (Fig 2a and 2b). For moderate amounts of applied current, the oscillator is pushed into an oscillatory limit cycle, and if sufficiently high amounts of current are applied, the system settles at a high fixed point.

For this system of coupled oscillators, brain regions (oscillators) can receive current from an external input (stimulation, i.e., *P* > 0) or from the activity of other brain regions to which they are connected. The global coupling parameter, *c*_5_ (see Methods), therefore serves to govern the global state of the system by regulating the overall amplitude of current transmitted between brain regions. For low values of *c*_5_, the system will fluctuate around the low fixed point, whereas for high values of *c*_5_, the system will transition into the oscillatory (limit cycle) regime. For a given structural connectivity, we can systematically increase the global coupling parameter and record the value at which the system transitions from the low fixed point to the oscillatory regime. Individual differences in structural connectivity can cause this transition to occur at different points in the parameter space (see S2 Fig), and we therefore use this point of transition to assess the sensitivity of our model to inter subject differences in the structural connectivity.

By measuring the point of oscillatory transition using structural connectivity matrices obtained from each of three scans for eight subjects, we see in Fig 2c that model dynamics are highly reproducible within scans of a single subject, but show variability across subjects. We quantify the within versus between subject reproducibility using the intraclass correlation coefficient (*ICC*). We see high reproducibility within scans of a single subject (Fig 2d, *ICC* = 0.826) and low reproducibility between different subjects (*ICC* = −0.006), which also corresponds to a low within subject variance (*V* = 0.0019) and high between subject variance (*V* = 0.0143). Due to the high within subject reproducibility across the three scans of each subject, the remaining findings are presented at the subject level by averaging the results over simulations derived from each of the three single subject scans.

### Regional controllability predicts the functional effect of stimulation

**Fig 3:**
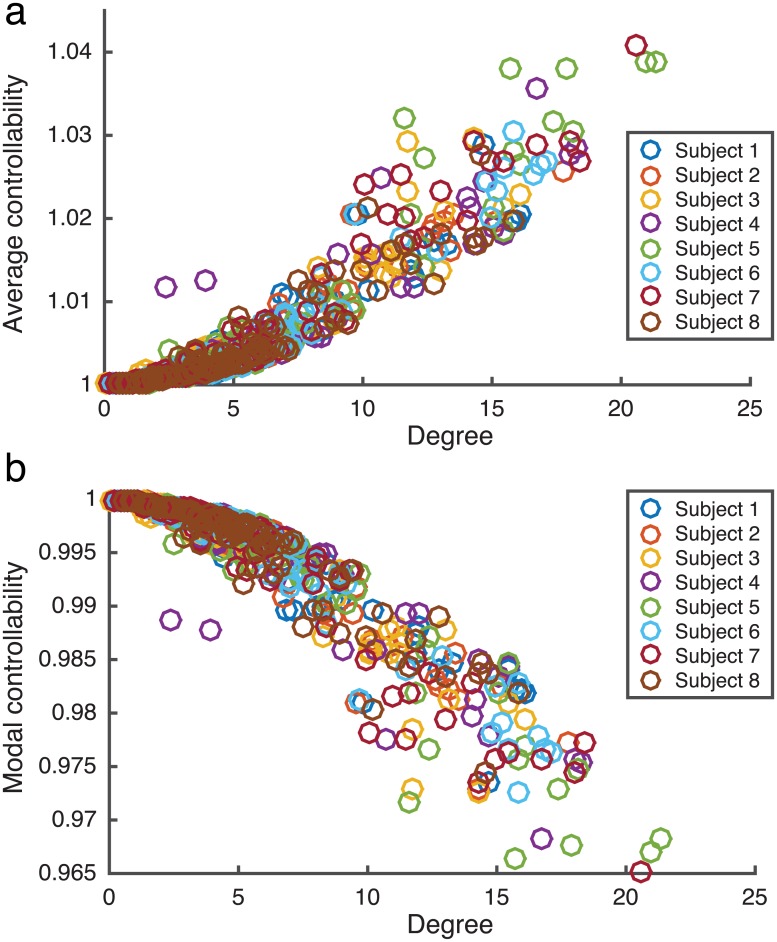
Linear regional controllability. (**a**) Average controllability plotted as a function of regional degree for each of the 83 brain regions. (**b**) Average controllability plotted as a function of regional degree. Controllability predictions were performed for each of the three scans for each subject and the data points reflect controllability and degree values averaged across scans.

In order to elucidate the role of regional stimulation to differentially control brain dynamics, we first turn to predictions made using linear network control theory [30]. Linear network control theory assumes a simplified linear model of network dynamics and computes controllability measures based upon the topological features of the structural network architecture. Here, we assess two different types of regional controllability derived from our structural brain networks: average controllability and modal controllability. Regions with high average controllability are capable of moving the system into many easy to reach states with a low energy input, whereas regions with high modal controllability can move the system into difficult to reach states but require a high energy input (see Methods for detailed descriptions of controllability measures and S1 Fig and S1 Text for their relationship to the steady state network response from regional stimulation with a constant current input). As previously described [30], we observe a strong correlation between regional degree and average controllability and a strong inverse correlation between regional degree and modal controllability that is robust across structural networks derived from all subjects (Fig 3).

We predict that brain regions with a high average controllability have the ability to impart large changes in network dynamics, easily moving the system into many nearby states. However, these predictions are made under the assumption of linear dynamics, and we know that the brain is in fact a highly nonlinear system. Our modeling approach allows us to directly test the validity of these linear controllability predictions in a nonlinear setting by systematically studying the effects of focal stimulation to brain regions and studying how the system moves.

**Fig 4:**
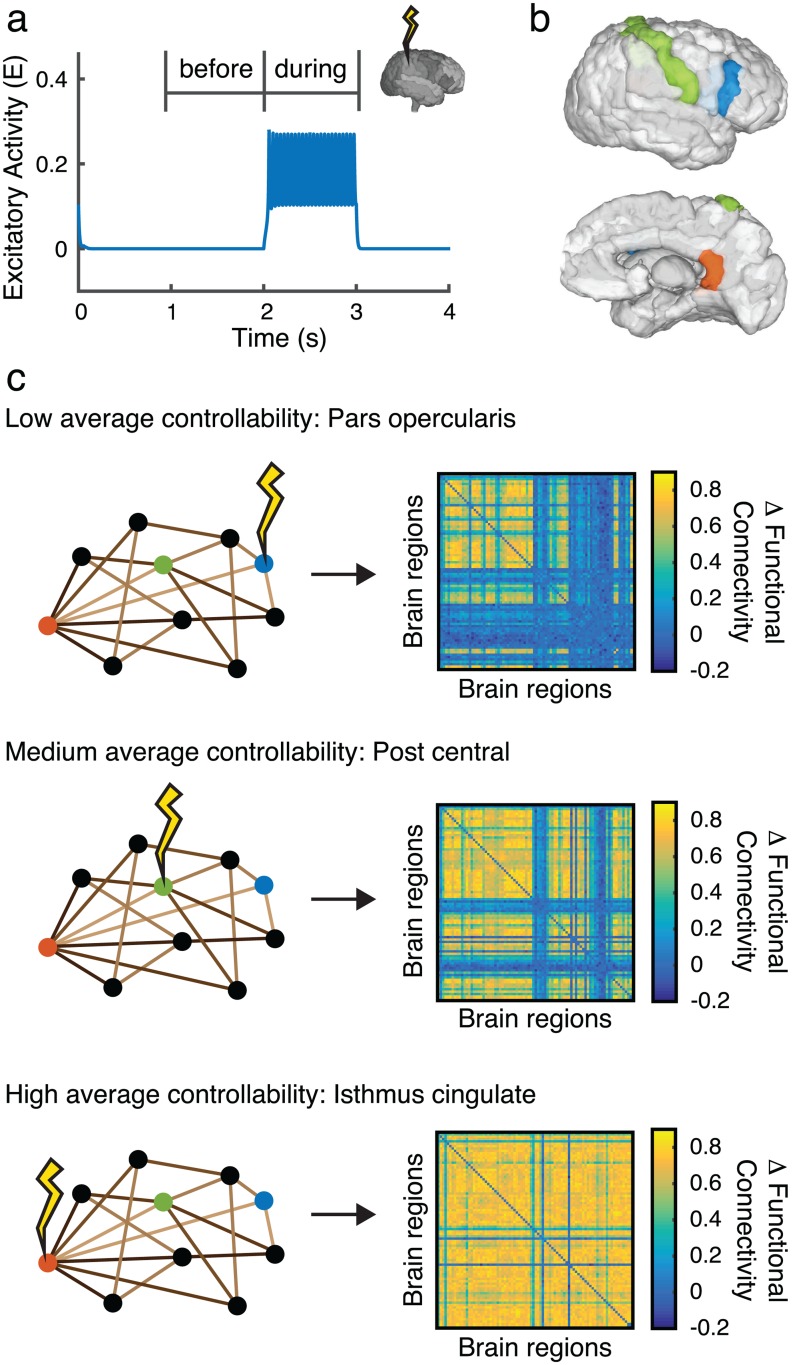
Regional stimulation. (**a**) Stimulation of a single region pushes the region from fluctuations around its low fixed point to the oscillatory state. (**b**) Example brain regions identified as having low average controllabllity (*pars opercularis*, blue), medium average controllability (*post central*, green) and high average controllability (*isthmus cingulate*, orange) (**c**) Simulation of example regions in panel *(b)* differentially move the system into new functional states. Stimulation applied to regions of high average controllability imparts more change in the functional brain state than stimulation applied to regions of low average controllability.

Using our computational model, we select the value of global coupling that places our brain model just before the transition to the oscillatory regime such that all brain regions are fluctuating near the low fixed point. We then select a single brain region and add an external stimulating current that brings the selected region into its oscillatory state (Fig 4a). We can quantify the changes in brain state due to this stimulation by computing the functional matrix of pairwise correlations between brain region dynamics during a period before stimulation occurs. We compare this pre-stimulation matrix to the functional matrix obtained during the stimulation period. By taking the difference between the functional brain state before and during regional stimulation, we measure the distance that the system moves. As expected, stimulation of low controllability regions produces smaller changes in the functional brain state than stimulation of high controllability regions (Fig 4b and 4c).

**Fig 5:**
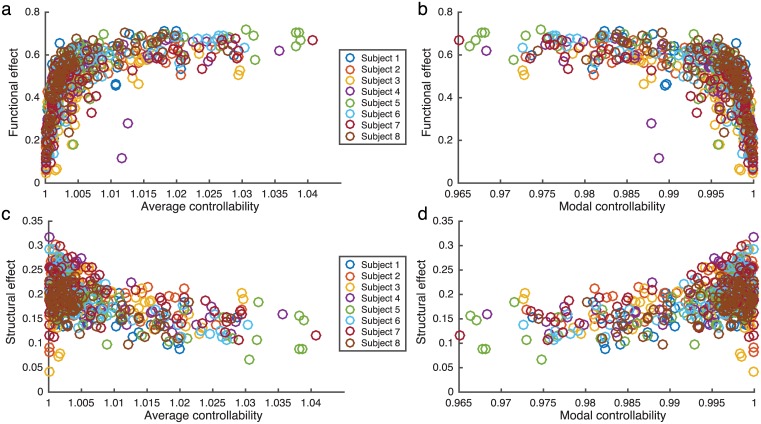
Functional effect of stimulation. (**a-b**) The functional effect of regional stimulation plotted as a function of the average *(a)* and modal *(b)* controllability for each of the 83 brain regions. (**c-d**) The structural effect of regional stimulation plotted as a function of the average *(c)* and modal *(d)* controllability for each of the 83 brain regions. Controllability predictions, simulations, and calculation of the functional and structural effects were performed for each of the three scans for each subject and the data points reflect values averaged over scans.

The results of systematically stimulating each brain region are shown in Fig 5 and S3 Fig. We quantify the overall change in brain state configuration by measuring the functional effect of regional stimulation: the absolute value of the pairwise change in functional connectivity, averaged over all brain region pairs. We observe that stimulation of regions with a high average controllability produce a large functional effect, while stimulation of regions with a high modal controllability result in a low functional effect (Fig 5a and 5b).

### Structural connectivity differentially constrains the effects of regional stimulation

We next ask how the underlying structural connectivity differentially constrains the functional effect of the stimulation for different brain regions. We quantify this structural constraint by calculating the structural effect on network dynamics, which measures the change in spatial correlation between the structural connectivity matrix and the functional brain state matrix before and during stimulation (Methods). Brain regions with a higher structural effect show a greater increase in similarity between structural and functional matrices when stimulated than regions with a lower structural effect. Thus, stimulation of these regions is more constrained by the underlying structural connectivity. As seen in Fig 5(c) and 5(d), the relationship between the structural effect and regional controllability is opposite that of the functional effect and controllability. Stimulation of regions with a high average controllability results in a smaller structural effect, while stimulation of regions with a high modal controllability results in a higher structural effect. The distributions of brain regions as a function of degree, average controllability, modal controllability, functional effect, and structural effect are shown in S4 Fig.

**Fig 6:**
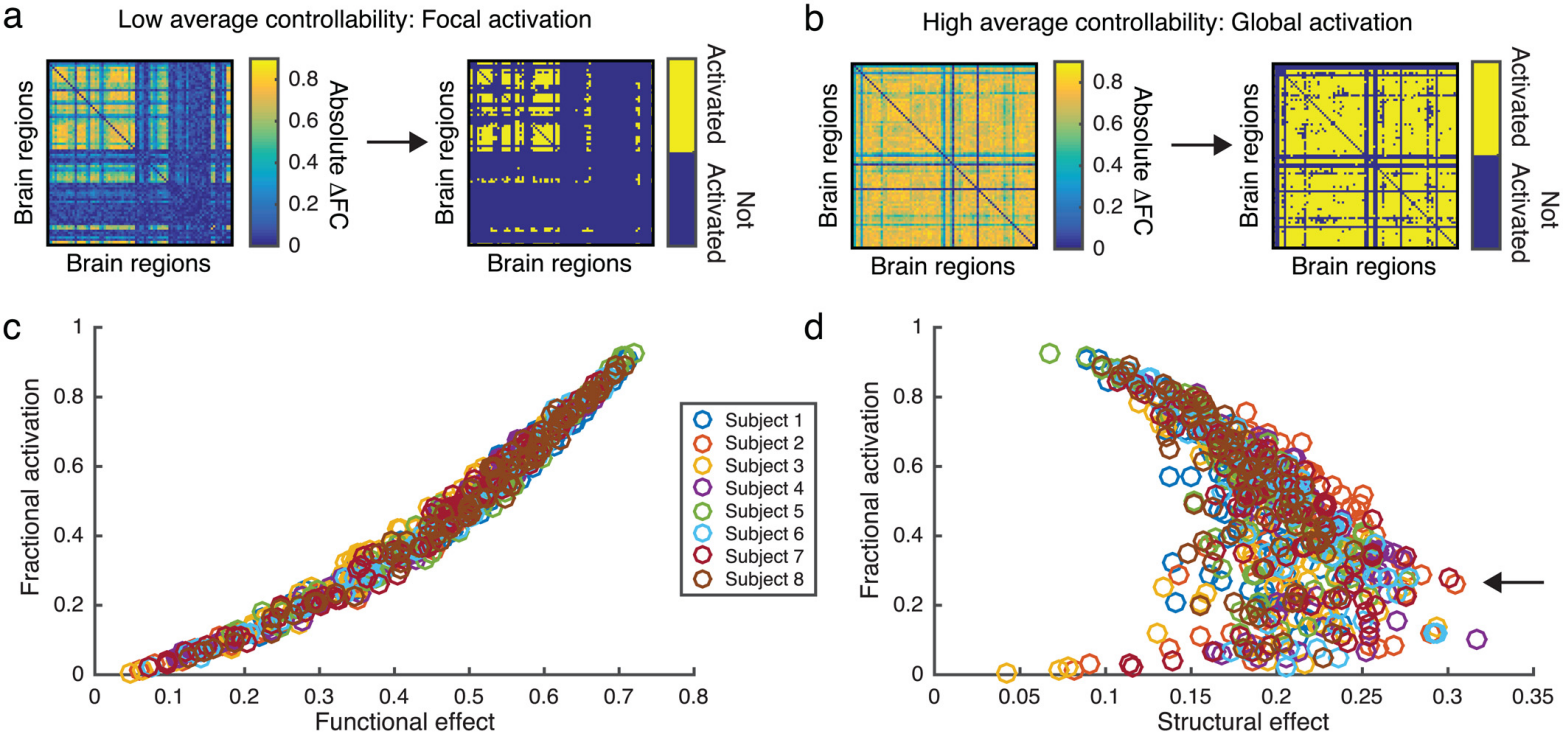
Fractional activation. (**a-b**) The absolute change in functional connectivity and resulting fractional activation shown for a threshold value of 0.6. *(a)* Example of stimulation applied to a region of low average controllability resulting in a focal effect on the resulting functional connectivity matrix. *(b)* Example of stimulation applied to a region of high average controllability resulting in a global effect on the resulting functional connectivity matrix. (**c**) The relationship between functional effect and fractional activation due to regional stimulation. We observe a high positive correlation between functional effect and fractional activation (Spearman’s *ρ* = .992, *p* ≪.001), indicating that a high functional effect corresponds to a global impact of stimulation while a low functional effect corresponds to a focal impact of stimulation. (**d**) The relationship between structural effect and fractional activation due to regional stimulation. Calculations of the functional and structural effects and fractional activation were performed for each of the three scans for each subject and the data points reflect values averaged over scans.

In order to better understand why stimulation of regions with a high average controllability easily moved the system (high functional effect) and were less constrained by the underlying structure (low structural effect), we quantified the spread of activation from the regional stimulation. Specifically, we asked if the stimulation of a given region induced focal or global changes in the brain state by calculating the fractional activation of the functional connectivity matrix. The fractional activation is given by the fraction of pairwise regions that experience a change in their functional connectivity value that is above a given threshold (yellow pixels in Fig 6a and 6b). If the stimulation of a brain region results in large changes that occur globally throughout the brain, the fractional activation will be high, but if the stimulation has only a focal effect, the fractional activation will be low.

As one might expect, we observe a strong positive correlation between the functional effect and the fractional activation (Fig 6c, Spearman’s *ρ* = .992, *p* ≪.001). Specifically, a large functional effect is due to a global effect of regional stimulation that pushes the system into a nearby state, while a small functional effect is due to a focal effect of regional stimulation that moves the system toward a more distant state. However, the relationship between the structural effect and fractional activation is more complex as seen in the crescent shaped curve of Fig 6d. A maximum structural effect occurs at the curve of the crescent (arrow in Fig 6d), where the effects of stimulation are neither focal or global. This result indicates that the underlying structural connections constrain the effects of stimulation the most in situations when regional stimulation impacts a moderately sized portion of the brain.

### Cognitive systems display varied roles in the structure-function landscape

**Fig 7:**
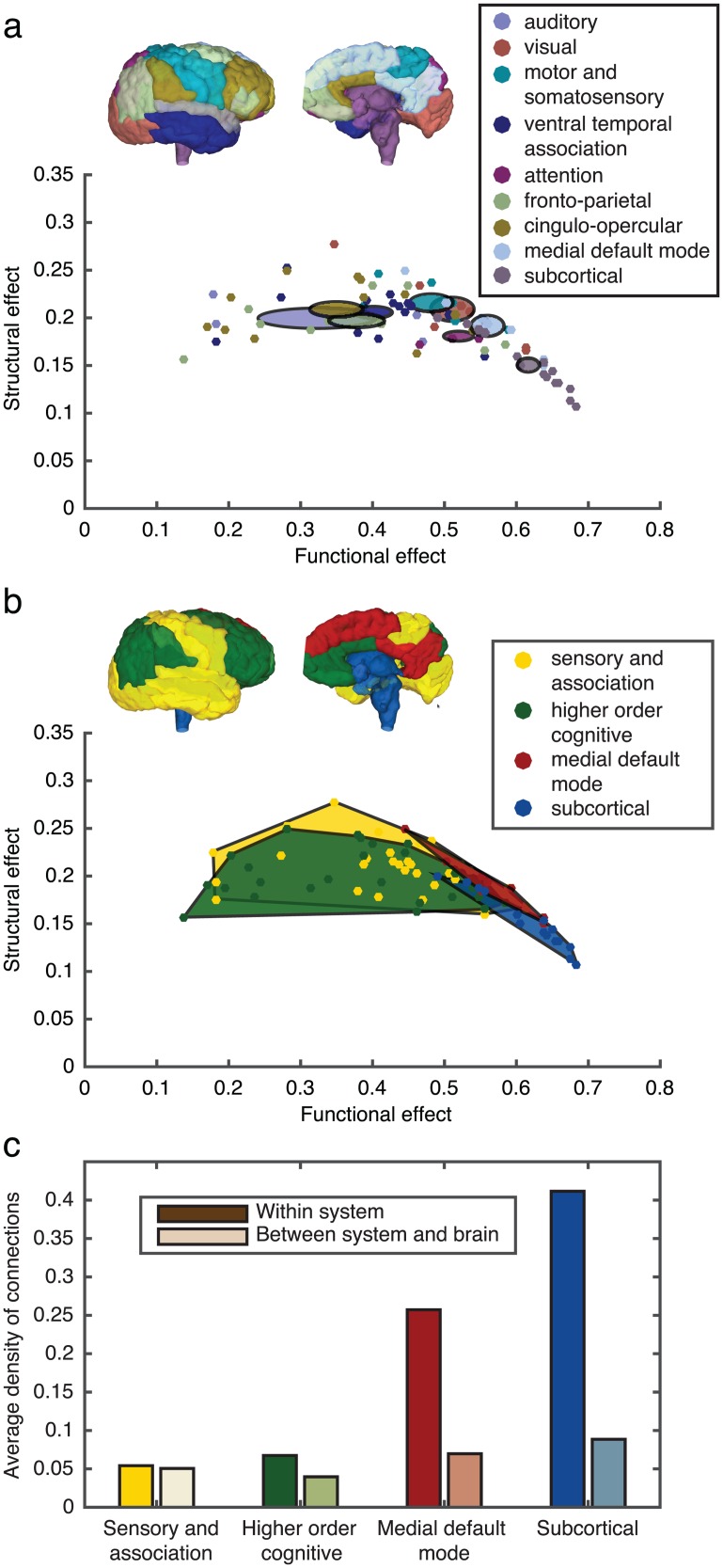
Structure-function landscape. (**a**) Structural and functional effect values for stimulation of individual brain regions sorted into 9 cognitive systems. Colored ellipses are centered on the mean structural and functional effects for a given cognitive system and the major and minor axis of the ellipse represent the standard error of the mean for the associated system. (**b**) Same as *(a)* but data is further course grained into 4 cognitive system types as in [36]. The colored regions indicate the convex hull surrounding the data points associated with the given system. Simulations, and calculations were performed for each of the three scans for each subject and the data points reflect values averaged over both scans and individuals. (**c**) Average density of connections within and between the four cognitive system types. Colors correspond to system assignments in *(b)* and dark shades represent the average density of connections between regions within a single cognitive system while light shades represent the average density of connections between regions within that system and regions outside of the system.

We next investigated the interplay between the structural and functional effects by examining the structure-function landscape (Fig 7). Stimulation of individual brain regions revealed a range of tradeoffs between structural and functional effect values, with some regions displaying a high functional effect but low structural effect and others displaying a high structural effect but moderate or low functional effect. We therefore asked if there was a relationship between the cognitive function associated with a brain region and its location in the structure-function landscape. Brain regions were assigned to one of nine cognitive systems (Fig 7 and Table A in S1 Text), eight of which were based on a data-driven clustering of functional brain networks [34] that group regions that perform similar roles across a diverse set of tasks, as well as a group of subcortical regions. Although in reality no region has a singular function, we use these system assignments as a pragmatic means to assess whether controllability diagnostics are differentially identified in distributed brain networks.

In Fig 7a, we can see that some cognitive systems cover a wide range of the structure-function landscape, whereas other systems tend to be more localized. Although the subcortical regions are well connected structurally, stimulation to any single subcortical region produces a functional effect that is less constrained by the underlying structural connections (low structural effect). In contrast, regions in other systems remain similarly constrained by the underlying structural connections, but vary in their ability to impart a functional effect on the system. Clusters of these nine cognitive systems emerge, suggesting that the parcellation scheme may be too fine-grained to understand the general organizing principles across the structure-function landscape; for example, there may be general network principles for sensory processing that are independent of modality (sight, sound, touch). Consequently, we created a more coarse-grained grouping of four cognitive systems (three functional and one structural) to examine broad-stroke differences among well-studied cognitive systems: sensory and association cortex, higher order cognitive, medial default mode network, and subcortical regions. As seen in Fig 7b, regions in the sensory and association cortex and higher order cognitive regions show a wide variation in their ability to impart a large functional effect when stimulated. Interestingly, although regions in the default mode network are similarly constrained by structure when compared to the sensory and association and higher order cognitive regions, regions within the default mode network consistently impart a large functional effect on the system. Stimulation of subcortical regions also results in a large functional effect, but these regions are less constrained by structure than those in the default mode and therefore these two systems occupy different spaces in the structure-function landscape (Fig 7b; two sample, two-dimensional Kolmogorov-Smirnov test, *p* = 0.0003). This separation in the structure-function landscape could reflect the fact that the default mode network represents a functionally defined system, whereas subcortical regions are functionally diverse despite being well connected structurally.

In our final analysis, we examined why the default mode and subcortical regions had a stronger functional effect, and interestingly, why the subcortical structures showed a lower structural effect. We hypothesized that differential properties of these four systems can be better understood by investigating the density of connections between brain regions within a system versus the density of connections between systems. Both the sensory and association cortex and the higher order regions are defined functionally and are composed of multiple distributed structural systems. They therefore, as a whole, have a low density of structural connections both within the system and between the system and the rest of the brain (Fig 7c). In contrast, subcortical regions form a highly connected structural subnetwork while remaining well connected to the rest of the brain. Thus, stimulation to these regions is globally distributed, resulting in a high functional effect. The medial default mode network also forms a well-connected subnetwork, but is less connected to the sensory and association cortex and higher order cognitive regions than the subcortical regions (see Table 1). While stimulation to regions in the default mode network results in a large functional effect, regions in this subnetwork remain more constrained by the underlying structure. This latter result is consistent with previous work showing that brain regions in the default mode network display the highest correlation between structural and functional connectivity [35]. These results capture a mechanism for the subcortical regions to strongly influence regions across cerebral cortex where the default mode has a strong but targeted functional effect within its network, which enables these regions to quickly adapt from rest to a wide variety of task states [30].

**Table 1:** Average density of connections between and within subnetworks of four cognitive systems.

	Sensory and association	Higher Order Cognitive	Default Mode	Subcortical
Sensory and association	0.054	0.025	0.059	0.094
Higher Order Cognitive	0.025	0.067	0.046	0.068
Default Mode	0.059	0.046	0.257	0.137
Subcortical	0.094	0.068	0.137	0.412

## Discussion

As neuromodulation is increasingly used to treat neurological disorders, it is essential to develop an understanding of the network-wide effects of focal stimulation. Such knowledge would directly inform the development of targeted protocols that effectively and efficiently maximize therapeutic benefits while minimizing the potential for adverse effects on brain dynamics and cognition. An initial step towards achieving this goal lies in the examination of neuroimaging data through the lens of linear network control theory, a mathematical framework that predicts highly controllable brain regions from the pattern of underlying structural connectivity. However, these techniques rely on the assumption that brain dynamics are linear, when in reality they are highly nonlinear.

Here, we developed a computational modeling approach to investigate whether the predictions of a region’s controllability drawn from a linear model could be validated in a nonlinear model. Specifically, we built data-derived structural connectivity matrices to computationally explore the effects of regional stimulation on network dynamics and functional states. Using this model, we confirmed predictions from linear control theory, showing that stimulation of high average controllability regions resulted in global activation that produced little change in the topology of the functional connectivity, underscoring their role in moving the brain to nearby states. Furthermore, we investigated the interplay between functional and structural effects of stimulation by examining how the global functional activity across brain regions was modulated by region-specific stimulation (a region’s functional effect) and whether the region’s structural connectivity accounted for its influence on the larger brain network (structural effect). We observed that the underlying network connectivity differentially constrained the effects of stimulation: regions of high average controllability (strongly connected hubs, most often associated with regions within the default mode network, see [30] for more discussion) displayed a high functional effect—meaning that they greatly increased the magnitude of functional connectivity—while regions of low average controllability (weakly connected areas [30]) did not. Yet, stimulation that led to larger changes in functional connectivity magnitude (functional effect) induced global changes in functional connectivity topology (fractional activation), moving the system towards easily reachable states as opposed to the more distant states accessed through focal activation due to stimulation of low controllability regions. Interestingly, when we parsed brain regions into cognitive systems, we found that stimulation of the medial default mode network showed both high structural and functional effects, differentiating it from other subnetworks by its ability to move the system while remaining influenced by the underlying network connectivity.

Perhaps one of the most striking observations from these data lies in the tradeoff between two competing consequences of stimulation: the magnitude of changes in functional connectivity, and the spatial specificity of changes in functional connectivity. We observed that stimulation to network hubs, predominantly located in default mode and subcortical structures [30, 31, 37], induces widespread increases in the magnitude of functional connectivity between brain regions. Yet, this broad impact is affected only at the expense of spatial specificity. In contrast, stimulation targeted to weakly connected areas (low average controllability) predominantly located in fronto-parietal regions [30], induces focal changes in functional connectivity. The differential impact of stimulation to these two strongly *versus* weakly connected regions suggests the possibility of two different classes of therapeautic interventions: (i) a broad reset, in which brain dynamics are globally altered, and (ii) a focal change, in which brain dynamics of a few regions are altered. While global alterations could be obtained through pharmacological interventions, the ability of focal regional stimulation to impart both global and focal alterations may offer mechanistic insights into the role of stimulation in distinguishing fine-scale differences between the concepts [38]*versus* broadly altering general cognitive processes [39, 40] or brain states [41].

The differences in the impact of stimulation to hubs *versus* non-hubs further supports the predictions of linear network control theory [42] and their recent applications to neuroimaging data [30]. In this prior work, theoretical insights from structural controllability [43] were used to make the prediction that network hubs—particularly in the default mode system—facilitate the movement of the brain to many easily reachable states. In contrast, weakly connected nodes of the network—particularly in cognitive control systems—were predicted to facilitate the movement of the brain to difficult-to-reach states. Our results confirm these predictions and offer further insights into the mechanisms of these control strategies. Specifically, easily reachable states are those that display patterns of functional connectivity that are very similar to those observed in the initial state, while difficult-to-reach states are those that display patterns of functional connectivity that are very different from those observed in the initial state.

In addition to these large-scale observations of network state change, we also probed the degree to which the pattern of functional connectivity (whether focal or global) was correlated with the pattern of structural connectivity. Prior work has focused largely on the relationship between resting state functional connectivity and structural connectivity [44–46], under the assumption that the brain’s resting baseline might be highly constrained by anatomy. However, structural connections likely constrain functional connectivity present in all brain states, irrespective of the cognitive process at play [30]. Indeed, a few recent studies have demonstrated the non-trivial relationships between individual differences in the pattern of structural connections and the observed functional connectivity across multiple cognitive states [47, 48]. Here we observe that the similarity between structural connectivity and observed functional connectivity depends significantly on the brain region that was stimulated. Critically, this relationship was not driven by the average controllability of the region. Along with prior links between average controllability and degree [30], these results suggest that structural constraints on stimulation-elicited functional connectivity can not easily be predicted by whether a region is a hub or a non-hub.

The relative independence of the functional and structural effects is particularly evident across large-scale cognitive systems. Indeed, we observe an inverted U-shaped curve between these two variables: systems that display a middling change in the magnitude of functional connectivity with stimulation tend to display functional connectivity patterns that are most reminiscent of the underlying structural connectivity. In contrast, systems that display a very large or very small change in the magnitude of functional connectivity with stimulation tend to display functional connectivity patterns that are very different from the structural connectivity. Intuitively, stimulation to hubs or non-hubs produces brain states that are far from those simply predicted by structural connectivity, and potentially thus far from normative [48]. It will be interesting in future to determine which brain states elicited by stimulation are consistent *versus* inconsistent with states observed in normative brain dynamics. Such a question is reminiscent of similar work in control theory identifying so-called *allowable* transitions [49, 50]. More broadly, an understanding of potential brain states elicited by stimulation is key to deploying stimulation in such a way as to maximize clinical benefit while minimizing pathological configurations of the network.

There are several important methodological considerations pertinent to this work. While this work represents an important step in characterizing the effects of stimulation and control in nonlinear brain networks, it should be noted that the nonlinear model of brain dynamics employed here, while biologically inspired, is a simplified mean-field model of neuronal dynamics. The sigmoidal transfer function introduced by Wilson and Cowan [32] simplifies the detailed spatial and temporal dynamics that underlie brain connectivity, and the fact that we have chosen to couple brain regions only through the excitatory populations reflects an assumption that most long range connections will be excitatory. Additionally, although noise is added to our model equations, we assume that all model parameters are identical between regions. While these assumptions impart limitations on the ability of the model to fully reproduce brain dynamics, our model represents a first step in understanding the role of brain topology in driving function, and we are encouraged by other studies showing that measures of network topology and similar modeling approaches depict a correlation between structural connectivity, simulated functional connectivity, and resting state fMRI functional connectivity [44, 51]. Hopefully, as experimental advances provide more insight into the nuances of brain connectivity, these features can be incorporated into future work, providing more insights into brain structure-function coupling and the role of stimulation and control.

Additionally, we have performed only a rough partitioning of the brain into regions and finer scales of regional partitioning could lead to greater distinction between regional roles as more subtle patterns of brain connectivity are revealed. While future work is necessary to confirm the effects of spatial resolution on models of targeted stimulation, in S5 Fig, we present the results of the relationships between controllability and the functional and structural effect for a single scan that has been parcellated into 234 regions. This figure shows that we see the same overall trends when parcellating the brain at a finer scale, indicating that the overall findings presented here still hold when partitioning the brain with greater spatial resolution.

Finally, an important finding of this study was that while we observed a high level of reproducibility in simulations run using connectivities derived from separate scans within a single subject, we observed variance across subjects in the range of coupling corresponding to the fixed point and oscillatory regimes of the model. While measures of controllability, functional, and structural effects collapsed to the same curves across subjects (Fig 5), the shifting of the oscillatory regime in the coupling parameter space (Fig 2c) indicates that the model is sensitive to variances in structural connectivities between individuals. Although the set of 8 subjects studied here is underpowered to study individual variation in connectivity and its impact on model performance, this encouraging result suggests the utility of such approaches to understand individual variability in structural connections and how it may change the functional effect of stimulation. This modeling approach provides a means to individualize stimulation protocols for personalized medical treatments or performance enhancements.

## Methods

### Ethics statement

All participants volunteered with informed consent in writing in accordance with the Institutional Review Board/Human Subjects Committee, University of California, Santa Barbara.

### Human DSI data acquisition and preprocessing

Diffusion spectrum images (DSI) were acquired from a total of 8 subjects in triplicate (mean age 27 ± 5 years, 2 female, 2 left handed) along with a *T*1 weighted anatomical scan at each scanning session [52]. DSI scans sampled 257 directions using a *Q*5 half shell acquisition scheme with a maximum *b* value of 5000 and an isotropic voxel size of 2.4mm. We utilized an axial acquisition with the following parameters: *TR* = 11.4s, *TE* = 138ms, 51 slices, FoV (231,231,123 mm).

DSI data were reconstructed in DSI Studio (dsi-studio.labsolver.org) using *q*-space diffeomorphic reconstruction (QSDR) [53]. QSDR first reconstructs diffusion weighted images in native space and computes the quantitative anisotropy (QA) in each voxel. These QA values are used to warp the brain to a template QA volume in MNI space using the SPM nonlinear registration algorithm. Once in MNI space, spin density functions were again reconstructed with a mean diffusion distance of 1.25 mm using three fiber orientations per voxel. Fiber tracking was performed in DSI Studio with an angular cutoff of 55°, step size of 1.0 mm, minimum length of 10 mm, spin density function smoothing of 0.0, maximum length of 400 mm and a QA threshold determined by DWI signal in the CSF. Deterministic fiber tracking using a modified FACT algorithm was performed until 100,000 streamlines were reconstructed for each individual.

Anatomical scans were segmented using FreeSurfer [54] and parcellated according to the Lausanne 2008 atlas included in the connectome mapping toolkit [31]. A parcellation scheme including 83 regions was registered to the B0 volume from each subject’s DSI data. The B0 to MNI voxel mapping produced via QSDR was used to map region labels from native space to MNI coordinates. To extend region labels through the gray/white matter interface, the atlas was dilated by 4mm. Dilation was accomplished by filling non-labeled voxels with the statistical mode of their neighbors’ labels. In the event of a tie, one of the modes was arbitrarily selected. Each streamline was labeled according to its terminal region pair.

### Construction of structural brain networks

We define structural brain networks by subdividing the entire brain into 83 anatomically distinct brain areas (network nodes) [55]. Consistent with prior work [37, 47, 48], we connect nodes by the number of white matter streamlines identified by a commonly used deterministic tractography algorithm described above [52] and normalized by the sum of the volumes of the nodes. This procedure results in sparse, weighted, undirected structural brain networks for each subject (*N* = 8) and each scanning session (*n* = 3), where network connections represent the density of white matter tracts between brain regions. The definition of structural brain networks based on tractography data in humans follows from our primary hypothesis that control features of neural dynamics are in part determined by the structural organization of the brain’s white matter tracts.

### Mathematical model of brain dynamics

We employ a data-driven, nonlinear model of brain dynamics that allows for the systematic study of the effects of stimulation to different nodes in the network. Brain regions (modeled by a single network node) are governed by Wilson-Cowan equations [32] representing the population-level activity of the *j*^*th*^ region as follows:

$$\tau \frac{dE_{j}}{dt}=-E_{j}\left(t\right)+\left(S_{e\_max}-E_{j}\left(t\right)\right)S_{e}\left(c_{1}E_{j}\left(t\right)-c_{2}I_{j}\left(t\right)+c_{5}\sum_{k}A_{jk}E_{k}\left(t-\tau_{d}^{k}\right)+P_{j}\left(t\right)\right)+\sigma w_{j}\left(t\right)$$

$$\tau \frac{dI_{j}}{dt}=-I_{j}\left(t\right)+\left(S_{i\_max}-I_{j}\left(t\right)\right)S_{i}\left(c_{3}E_{j}\left(t\right)-c_{4}I_{j}\left(t\right)\right)+\sigma v_{j}\left(t\right)$$

where *E*(*t*)/*I*(*t*) represent the firing rate of the excitatory/inhibitory population respectively, *τ* = 8 ms is a time constant, *P*(*t*) is an external stimulus parameter, and *w*_*j*_(*t*) and *v*_*j*_(*t*) are drawn from a standard normal distribution and act as additive noise to the system with *σ* = 0.00001. Regions are coupled through the excitatory population with a connectivity matrix **A** derived from a single scan of an individual subject’s tractography data, parcellated to give a total of 83 brain regions, and normalized by region size. Delays, *τ*_*d*_, between regions are calculated as a function of physical distance between identified brain regions, assuming a transmission velocity of 10 m/s (range *τ*_*d*_ = 0.8 − 14.8 ms). The transfer function is given by the sigmoidal function

$$S_{e/i}\left(x\right)=\frac{1}{1+e^{\left(-a_{e/i}\left(x-\theta_{e/i}\right)\right)}}-\frac{1}{1+e^{a_{e/i}\theta_{e/i}}}.$$

Model constants are set as in [32] to be *c*_1_ = 16, *c*_2_ = 12, *c*_3_ = 15, *c*_4_ = 3, *a*_*e*_ = 1.3, *a*_*i*_ = 2, *θ*_*e*_ = 4, *θ*_*i*_ = 3.7. These parameter choices imply that an uncoupled oscillator has three states: a low fixed point, limit cycle, and high fixed point. For a single oscillator, increasing the current input into the oscillator, *P*(*t*), will allow it to transition between the three states. In our model of coupled oscillators, the global coupling parameter, *c*_5_, serves as an additional mechanism for allowing the system to transition between these states for fixed values of *P*(*t*). To simulate brain activity, we set *P*(*t*) = 0 for all regions, and we model stimulation to single brain region, *j*, by setting *P*_*j*_(*t*) = 1.25. This value of *P* implies that an uncoupled oscillator will enter into limit cycle activity with a frequency near 20 Hz which is inline with the biological range for oscillatory brain activity. All simulations are allowed to initially stabilize for 1s before analysis, and the temporal dynamics of the *j*^*th*^ brain region are given by the firing rate of the excitatory population, *E*_*j*_(*t*).

### Evaluating oscillatory transition parameters

In order to asses the point in the parameter space at which the system switched from the low fixed point to the oscillatory regime, we ran 1s simulations in which no stimulating current was applied (*P* = 0) for increasing values of the global coupling parameter (*c*_5_ = 1.0 to *c*_5_ = 1.5 in step sizes of 0.05). The average firing rate of the excitatory population for each region was recorded as a function of the global coupling parameter and is shown for stimulations using a single scan from two separate subjects in S2 Fig. At a certain value of the global coupling parameter that varied between scans and subjects, we see a sudden increase in the firing rate across most regions, indicating the transition of the system from the fixed point to the oscillatory regime. We use the value of *c*_5_ at which this transition occurs to assess the reproducibility and variability within and between subjects. For all simulations assessing the impact of regional stimulation, the value of *c*_5_ immediately before this transition was used.

### Inter- and intra-subject reproducibility and variability

Inter- and intra-subject reproducibility were calculated using the intraclass correlation coefficient (ICC), and assuming a random effect model as in [56]. The between subject ICC is estimated using the mean squares (MS) obtained by applying ANOVA with the subject as the factor to the value of global coupling at which the system switches to the oscillator state obtained as described above:

$$ICC_{B}=\frac{J(SMS-EMS)}{J*SMS+I*RMS+(IJ-I-J)EMS}.$$ (1)

Similarly, the within subject ICC is given by

$$ICC_{W}=\frac{I(RMS-EMS)}{J*SMS+I*RMS+(IJ-I-J)EMS},$$ (2)

where *I* is the number of subjects, *J* is the number of scans, *SMS* is the MS between scans, *RMS* is the MS between subjects, and *EMS* is the MS due to error.

The between subject variability was defined to be the variance of the mean value of the transition point obtained for each subject, and the within subject variability was defined to be the within subject variance, averaged across all subjects.

### Linear network control theory

To study the theoretical ability of a certain brain region to influence other regions in arbitrary ways we adopt the control theoretic notion of *controllability*. Controllability of a dynamical system refers to the possibility of driving the state of a dynamical system to a specific target state by means of an external control input [57]. As in [30], we employ a simplified noise-free linear discrete-time and time-invariant network model:

$$x\left(t+1\right)=Ax\left(t\right)+B_{K}u_{K}\left(t\right),$$ (3)

where **x** describes the state of brain regions over time, **A** is a normalized structural connectivity matrix derived from tractography data as described above. In S1 Text contains a discussion of the method used to normalize matrices across subject data sets. The input matrix $B_{K}$ identifies the control points, and $u_{K}$ denotes the control strategy.

Classic results in control theory ensure that controllability of the network Eq (3) from the set of network nodes K is equivalent to the controllability Gramian $W_{K}$ being invertible, where

$$W_{K}=\sum_{\tau =0}^{\infty }A^{\tau }B_{K}B_{K}^{T}A^{\tau }.$$ (4)

We utilize this framework to choose control nodes one at a time, and thus the input matrix *B* in fact reduces to a one-dimensional vector.

We examine 2 diagnostics of controllability utilized in the network control literature: *average controllability* and *modal controllability* [30]. Average controllability of a network equals the average input energy from a set of control nodes and over all possible target states [58, 59]. As in [30], we adopt Trace(*W*_*K*_) as a measure of average controllability. Regions with high average controllability are, on average, most influential in the control of network dynamics over all nearby target states with least energy. Modal controllability refers to the ability of a node to control each evolutionary mode of a dynamical network [60], and can be used to identify states that are difficult to control from a set of control nodes. Modal controllability is computed from the eigenvector matrix *V* = [*v*_*ij*_] of the network adjacency matrix **A**. As in [30], we define the modal controllability to be $\phi_{i}=\sum_{j=1}^{N}\left(1-\lambda_{j}^{2}\left(A\right)\right)v_{ij}^{2}$, providing a scaled measure of the controllability of all *N* modes *λ*_1_(*A*),…,*λ*_*N*_(*A*) from the brain region *i*. Regions with high modal controllability are able to control all the dynamic modes of the network, and hence they can drive the dynamics towards hard-to-reach configurations.

The stimulation paradigm that we study is a constant input over a short period of time, whereas the controllability metrics discussed above assume a more general time-varying input. We treat this constant current stimulation as a a simple approximation of the more general stimulation paradigm traditionally studied in the control literature, and therefore use average and modal controllability to assess control architecture. Because our stimulation paradigm is given by a constant input, one can also examine the steady state response of the network. In S1 Text and S1 Fig, we study the more constrained steady state response of the network, and demonstrate that our approximation presented here is an accurate one. However, it should be noted that the statistics we use here (average/modal controllability) may not always be an adequate approximation of the steady state response of a network, and therefore care should be taken in using these statistics in other studies without first demonstrating their consistency with the steady state response statistics.

### Quantifying functional brain states

We quantify brain states by calculating the pairwise maximum normalized cross-correlation [61, 62] between the firing rate of the excitatory populations, *E*_*i*_(*t*) and *E*_*j*_(*t*), for brain regions *i* and *j*. All calculations are performed using a 1 s window and a maximum lag of 250 ms.

### Functional effect of stimulation

Simulations of neural dynamics are first allowed to stabilize for 1s to reach the stable activity from the global coupling parameter and then the remaining time is divided into two parts: a stimulation free period of 1s, followed by a 1s period of stimulation. During the stimulation period, a single region, *s*, is selected and a stimulus is applied to this region by setting *P*_*s*_(*t*) = 1.25, while *P*(*t*) = 0 for all other regions. Functional brain states are computed separately for the stimulation-free (‘before’ in Fig 4) and stimulation (‘during’ in Fig 4) periods. We assess the pairwise change in functional brain states by subtracting the correlation values obtained in the stimulation-free time window before the stimulation is applied from the correlation values obtained in the time window during the stimulation. We then measure the average change in functional brain states, termed the *functional effect*, as the absolute value of this difference averaged over all region pairs. The greater the deviation of the functional effect from zero, the greater the effect of stimulation on brain state reconfiguration.

### Structural effect on network dynamics

In order to assess how the structural brain connectivity constrains network dynamics, we calculate the similarity, defined by the 2-dimensional correlation coefficient, between the structural connectivity matrix used as a basis of the simulation and the functional matrix describing the resultant brain state. This calculation is done first using the initial functional brain state before stimulation, and then using the functional brain state during the stimulation period. Stimulation of a single region increases the similarity between the structural connectivity and functional brain state, and we quantify this increase, termed the *structural effect*, by subtracting the correlation obtained before stimulation from that obtained during stimulation. Thus, a high structural effect indicates that the underlying connectivity structure constrains the functional effects of stimulation to this region.

### Fractional activation

The fractional activation due to the stimulation of a single brain region is defined to be the fraction of pairwise regions that experience a change in their functional connectivity value that is above a given threshold. The change in functional connectivity is calculated as described above to calculate the *functional effect* by assessing the absolute change in pairwise correlation values before and during regional stimulation. If the stimulation of a brain region results in large changes that occur globally throughout the brain, the fractional activation will be high, but if the stimulation has only a focal effect, the fractional activation will be low. Here, we report findings using a threshold value of 0.6, however, results were similar across a range of thresholds (S6 Fig).

## Supporting Information

S1 Fig Steady state response and functional effect. The functional effect resulting from regional stimulation plotted as a function of (**a**) the largest steady state value (**b**) the average steady state value.
(EPS)

S2 Fig Transition to oscillatory regime. (**a-b**) Examples of the transition to the oscillatory regime in simulations from a single scan obtained from two different subjects. In (a) the transition occurs for a global coupling value of *c*_5_ = 1.4 whereas in (b) the transition occurs for *c*_5_ = 1.25.
(EPS)

S3 Fig ‘Zoomed-in’ functional and structural effect as a function of controllability measures. Same as Fig 5 in the main manuscript, except that the x-axis zooms-in on only small values of average controllability and large values of modal controllability.
(EPS)

S4 Fig Distributions of brain regions for observed measures. Distributions of brain regions as a function of (**a**) degree, (**b**) average controllability, (**c**) modal controllability, (**d**) functional effect, and (**e**) structural effect.
(EPS)

S5 Fig Effect of stimulation for a parcellation with 234 brain regions. (**a-b**) The functional effect of regional stimulation plotted as a function of the average *(a)* and modal *(b)* controllability for each of the 234 brain regions from a single scan. (**c-d**) The structural effect of regional stimulation plotted as a function of the average *(c)* and modal *(d)* controllability for each of the 234 brain regions from a single scan. Results are similar to those presented in the main manuscript for the coarser parcellation with only 83 brain regions.
(EPS)

S6 Fig Fractional activation for varied threshold values. (**a-b**) The fractional activation calculated using a threshold value of 0.2 shown as a function of Functional Effect (a) and Structural Effect (b). (**c-d**) The same as (a-b) but using a threshold value of 0.4. These results are comparable to that shown in Fig 6c and 6d of the main manuscript.
(EPS)

S1 Text The text for the Supporting Information can be found in the file Muldoon_SI.pdf. (PDF)
