# Supplementary material for: Stimulation-Based Control of Dynamic Brain Networks
Source: PLoS Comput Biol. 2016 Sep 9;12(9):e1005076. doi: 10.1371/journal.pcbi.1005076 (PMC5017638; doi:10.1371/journal.pcbi.1005076)
Supplement: S1 Text — (PDF) [file pcbi.1005076.s007.pdf]

Supporting Information for *Stimulation-Based Control of Dynamic Brain Networks*

Sarah Feldt Muldoon[1,2,3], Fabio Pasqualetti[4], Shi Gu[1,5], Matthew Cieslak[6], Scott T. Grafton[6], Jean M. Vettel[2,6,1], Danielle S. Bassett[1,7,*]

1 Department of Bioengineering, University of Pennsylvania, Philadelphia, PA 19104
2 US Army Research Laboratory, Aberdeen Proving Ground, MD 21005
3 Department of Mathematics and Computational and Data-Enabled Science and Engineering Program, University at Buffalo, SUNY, Buffalo, NY 14260
4 Department of Mechanical Engineering, University of California, Riverside, CA 92521
5 Applied Mathematics and Computational Science Graduate Program, University of Pennsylvania, Philadelphia, PA 19104
6 Department of Psychological and Brain Sciences, University of California, Santa Barbara, Santa Barbara, CA 93106
7 Department of Electrical and Systems Engineering, University of Pennsylvania, Philadelphia, PA 19104

*** dsb@seas.upenn.edu**

Normalization of structural matrices across subjects

When doing controllability calculations, we must ensure that the structural adjacency matrix describing the tractography connectivity is Schur stable. In Gu et al. [1], this was obtained by normalizing each structural matrix by one plus its largest singular value. Because each matrix effectively received a slightly different normalization factor, regional controllability results were ranked in order to perform averaging across scans and subjects. To avoid the loss of information that results from ranking the data, we instead employed a different form of normalization. We first calculated the maximum eigenvalue for each structural matrix in the data set. From this pool of maximum eigenvalues, we then selected the maximum value, and divided all structural matrixes by two times this quantity. This ensures Schur stability and allows us to compare directly compare regional controllability values obtained from different structural matrices. The controllability results presented in this paper therefore represent the resultant numerical values of regional controllability calculations, as opposed to the ranked values presented in Gu et al [1].

Average controllability and the steady state response

In linear network control theory, the controllability of a network refers to the possibility of altering the configuration of the network nodes via external stimuli and in a predictable way. To quantify the degree of controllability of a network, we first

model the dynamical interaction among network nodes by means of a discrete-time, linear, time-invariant system:

$$x(t+1) = Ax(t) + B_K u(t).$$

In the equation above, x is a vector containing the states of the network nodes, $A = A^{\mathsf{T}}$ is a (stable) weighted adjacency matrix of the network, u is the external control signal, and B_K identifies the control nodes; see also [1, 2].

In our simulations, we use a constant input current to stimulate brain regions, and therefore are interested in the steady state response of the network. With a constant control input, the network steady state is

$$x_{\text{steady}} = (I - A)^{-1} B_K u_{\text{constant}},$$

where u_{constant} is the value of the constant input. Thus, the steady state effect of a constant input to the i-th region is characterized by the i-th column of the matrix $(I - A)^{-1}$. The largest value of the i-th column gives steady state response of the region maximally effected by the input, while the average of the i-th column gives the average steady state effect over all regions.

The use of a constant input as regional stimulation is a special case of the more general paradigm of a time-varying input normally used to define network control statistics such as those used in the main manuscript (average/modal controllability). We therefore would like to relate this steady state response to the average controllability which describes the more general paradigm. The degree of controllability of a network can be quantified in different ways [2], but in this paper, we use the classical definition of the Controllability Gramian, that is,

$$W_K = \sum_{\tau=0}^{\infty} A^{\tau} B_K B_K^T A^{\tau},$$

and measure the *average* degree of controllability as $\text{Trace}(W_K)$, which has a specific system theoretic interpretation [1–3]. Moreover, notice that

$$\begin{aligned}
\text{Trace}(W_K) &= \text{Trace}\left(\sum_{\tau=0}^{\infty} A^{\tau} B_K B_K^T A^{\tau}\right) \\
&= \sum_{\tau=0}^{\infty} \text{Trace}\left(A^{2\tau} B_K B_K^{\mathsf{T}}\right) \\
&= \text{Trace}\left(\sum_{\tau=0}^{\infty} A^{2\tau} B_K B_K^{\mathsf{T}}\right) \\
&= \text{Trace}\left((I - A^2)^{-1} B_K B_K^{\mathsf{T}}\right) \\
&= \sum_{i \in K} (I - A^2)^{-1}_{ii}.
\end{aligned}$$

In other words, the controllability degree with control nodes K equals the sum of the diagonal entries of $(I - A^2)^{-1}$ indexed by K. Because of the normalization of the adjacency matrix adopted in this work, it can be verified that

$$(I - A)^{-1} \approx (I - A^2)^{-1},$$

so that the average controllability information can be reconstructed from the steady state response matrix $(I - A)^{-1}$. Specifically, for stimulation of a single region, the

largest entry of the i-th column of the steady state matrix will be approximately equal to the average controllability.

As seen in S1 Fig (a), when we plot the functional effect of stimulation as a function of the largest steady state value, we do indeed reproduce the results of Fig. 5a. Additionally, we see a similar result when plotting the functional effect of stimulation for the average steady state value (S1 Fig (b)). Therefore, in the main manuscript, we present our findings in terms of the more general regional controllability values instead of the steady state response, which also allows for comparison with previous work using these measures to study the properties of structural brain networks [1]. However, it should be noted that the average/modal controllability may not always be an adequate approximation of the steady state response of a network, and therefore care should be taken in using these statistics in other studies without first demonstrating their consistency with the steady state response statistics.

Mapping regions to cognitive systems

Similar to the assignment of brain regions in Gu et al. [1] and inspired by Power et al. [4], we initially assigned each of the 83 brain regions to one of 9 cognitive systems. Our only divergence from Gu et al. was the creation of a "ventral temporal association" category to bin perceptual regions associated with invariant object representations and multisensory activation. For further analysis, this assignment was coarse grained into 4 cognitive systems [5]. The placement of each region into each cognitive system is summarized in Table A.

References

1. Gu S, Pasqualetti F, Cieslak M, Telesford QK, Yu AB, Kahn AE, et al. Controllability of structural brain networks. Nature Communications. 2015;6:8414.

2. Pasqualetti F, Zampieri S, Bullo F. Controllability metrics, limitations and algorithms for complex networks. IEEE Trans Control Netw Syst. 2014;1:40–52.

3. Kailath T. Linear Systems. Prentice Hall; 1980.

4. Power JD, Cohen AL, Nelson SM, Wig GS, Barnes KA, Church JA, et al. Functional Network Organization of the Human Brain. Neuron. 2011 Nov;72(4):665–678.

5. Gu S, Satterthwaite TD, Medaglia JD, Yang M, Gur RE, Gur RC, et al. Emergence of system roles in normative neurodevelopment. Proceedings of the National Academy of Sciences of the United States of America. 2015 Nov;112(44):13681–13686.

Region Name	9 system assignment	4 system assignment
Lateral Orbitofrontal	attention	higher order cognitive
Pars Orbitalis	cingulo-opercular	higher order cognitive
Frontal Pole	fronto-parietal	higher order cognitive
Medial Orbitofrontal	fronto-parietal	higher order cognitive
Pars Triangularis	fronto-parietal	higher order cognitive
Pars Opercularis	cingulo-opercular	higher order cognitive
Rostral Middle Frontal	cingulo-opercular	higher order cognitive
Superior Frontal	medial default mode	medial default mode
Caudal Middle Frontal	fronto-parietal	higher order cognitive
Precentral	motor and somatosensory	sensory and association
Paracentral	motor and somatosensory	sensory and association
Rostral Anterior Cingulate	cingulo-opercular	higher order cognitive
Caudal Anterior Cingulate	cingulo-opercular	higher order cognitive
Posterior Cingulate	medial default mode	medial default mode
Isthmus Cingulate	medial default mode	medial default mode
Post Central	motor and somatosensory	sensory and association
Supramarginal	cingulo-opercular	higher order cognitive
Superior Parietal	attention	higher order cognitive
Inferior Parietal	fronto-parietal	higher order cognitive
Precuneus	medial default mode	medial default mode
Cuneus	visual	sensory and association
Pericalcarine	visual	sensory and association
Lateral Occipital	visual	sensory and association
Lingual	visual	sensory and association
Fusiform	ventral temporal association	sensory and association
Parahippocampal	ventral temporal association	sensory and association
Entorhinal Cortex	ventral temporal association	sensory and association
Temporal Pole	ventral temporal association	sensory and association
Inferior Temporal	ventral temporal association	sensory and association
Middle Temporal	ventral temporal association	sensory and association
Bank of the Superior Temporal Sulcus	ventral temporal association	sensory and association
Superior Temporal	auditory	sensory and association
Transverse Temporal	auditory	sensory and association
Insula	fronto-parietal	higher order cognitive
Thalamus	subcortical	subcortical
Caudate	subcortical	subcortical
Putamen	subcortical	subcortical
Pallidum	subcortical	subcortical
Nucleus Accumbens	subcortical	subcortical
Hippocampus	subcortical	subcortical
Amygdala	subcortical	subcortical
Brainstem	subcortical	subcortical

Table A. Assignment of brain regions to cognitive systems.
